# Customizable Fabrication of Photothermal Microneedles with Plasmonic Nanoparticles Using Low-Cost Stereolithography Three-Dimensional Printing

**DOI:** 10.1021/acsabm.4c00411

**Published:** 2024-06-15

**Authors:** Jill Ziesmer, Isabel Sondén, Justina Venckute Larsson, Padryk Merkl, Georgios A. Sotiriou

**Affiliations:** Department of Microbiology, Tumor and Cell Biology, Karolinska Institutet, Stockholm SE-171 77, Sweden

**Keywords:** Ag, silver nanoaggregates, flame spray pyrolysis, plasmonic coupling, antibacterial, skin infections

## Abstract

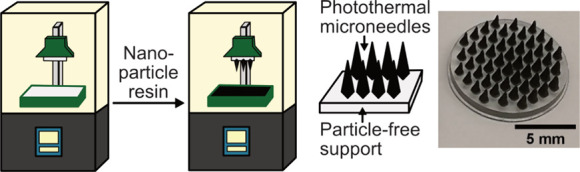

Photothermal microneedle (MN) arrays have the potential to improve the treatment of various skin conditions such as bacterial skin infections. However, the fabrication of photothermal MN arrays relies on time-consuming and potentially expensive microfabrication and molding techniques, which limits their size and translation to clinical application. Furthermore, the traditional mold-and-casting method is often limited in terms of the size customizability of the photothermal array. To overcome these challenges, we fabricated photothermal MN arrays directly via 3D-printing using plasmonic Ag/SiO_2_ (2 wt % SiO_2_) nanoaggregates dispersed in ultraviolet photocurable resin on a commercial low-cost liquid crystal display stereolithography printer. We successfully printed MN arrays in a single print with a translucent, nanoparticle-free support layer and photothermal MNs incorporating plasmonic nanoaggregates in a selective fashion. The photothermal MN arrays showed sufficient mechanical strength and heating efficiency to increase the intradermal temperature to clinically relevant temperatures. Finally, we explored the potential of photothermal MN arrays to improve antibacterial therapy by killing two bacterial species commonly found in skin infections. To the best of our knowledge, this is the first time describing the printing of photothermal MNs in a single step. The process introduced here allows for the translatable fabrication of photothermal MN arrays with customizable dimensions that can be applied to the treatment of various skin conditions such as bacterial infections.

## 1 Introduction

Photothermal therapy (PTT) involves the use of electromagnetic radiation and photothermal agents to improve medical therapy via heat. PTT has been widely described for the treatment of various cancers through thermal ablation of malignant cells.^1−3^ The ability to weaken cells or induce cell death can also be beneficial for antibacterial treatments and has recently gained attention.^4−6^ Although topical application of photothermal formulations may be successful against superficial infections, expanding the PTT intradermally may allow for treatment of deeper cutaneous bacterial infections.^7^

Microneedle (MN) arrays have emerged as promising tools to extend topical dermatotherapies to intradermal delivery in a minimally invasive manner.^8−10^ Such arrays consist of multiple needles in conical or pyramidical shapes generally with heights ranging from 10 to 2000 μm which can pierce the *stratum corneum* without causing the pain associated with hypodermic needle injections.^11^ MN arrays are often used to deliver a pharmaceutical drug into or across the skin; however, photothermal agents have also recently been delivered to the skin via MNs.^12^ Providing PTT through MN arrays has improved the treatment of skin cancers^13−15^ and bacterial infections^16^ and accelerated wound healing.^17^ However, photothermal MN arrays are produced with rather costly photothermal agents such as gold nanorods^14,18−20^ or with photosensitizers which are subject to photobleaching.^13,15,21,22^ Furthermore, the photothermal MN arrays are fabricated via micromolding, which requires a multistep protocol.^9^ Micromolding usually relies on the microfabrication of a metal master template via photolithography, which is time-consuming and expensive due to long processing times and the need for cleanroom facilities.^23^ Most importantly, micromolding limits the shape and dimensions of the fabricated MN array, precluding patient-specific treatments and the possibility to selectively treat only diseased skin areas. Such shortcomings can be a limiting factor when trying to translate new medical devices into the clinics.

To overcome the problems associated with micro- and nanolithography production of MNs, additive manufacturing has become a versatile tool to manufacture customizable MN arrays.^24−26^ Typically, a prototype of the MNs is designed with a computer-aided design (CAD) software and subsequently sliced and exported as a printer-specific file, which is uploaded to the printer. 3D-printers based on vat photopolymerization have successfully been used to manufacture high-resolution MNs in a reproducible fashion, and especially stereolithography (SLA) printers are of interest due to their affordability, high resolution, and ease of use.^26^ Krieger et al. recently utilized a commercially available laser-based SLA-printer for the rapid fabrication of MNs with sharp tips, which were successfully used for the creation of a MN mold.^27^ Such MN molds can subsequently be used to fabricate MN arrays that deliver photothermal cargo. For example, El-Sayed et al. employed an SLA-printer to print MN templates and followed the mold-and-casting method to fabricate MN arrays loaded with gold nanorods.^28^ Alternatively, a photothermal or pharmaceutical agent can also be coated onto 3D-printed MN arrays via inkjet printing.^29^ However, these studies rely on a multistep production method, which reduces the fabrication speed and increases the costs of the final photothermal MN array.

We present here for the first time the manufacturing of photothermal MN arrays in a single 3D-print using a commercial, low-cost SLA-printer. The photothermal agent we incorporate into the MNs are flame-made plasmonic photothermal Ag/SiO_2_ (2 wt % SiO_2_) nanoaggregates with tuned absorption to the near-infrared (NIR) region rendering them suitable for application in human tissue.^30^ We optimized the dispersion of the nanoaggregates in the resin by comparing three different mixing protocols. Furthermore, we studied the customizability of the printed photothermal MN arrays, analyzed their mechanical strength, and measured their photothermal effect both *in vitro* and *ex vivo* in skin. Finally, we demonstrate the potential to apply such MN arrays for the treatment of infections via *in vitro* antibacterial studies.

## 2 Results and Discussion

### 2.1 Fabrication of 3D-Printed, Photothermal MN Arrays

**Figure 1:**
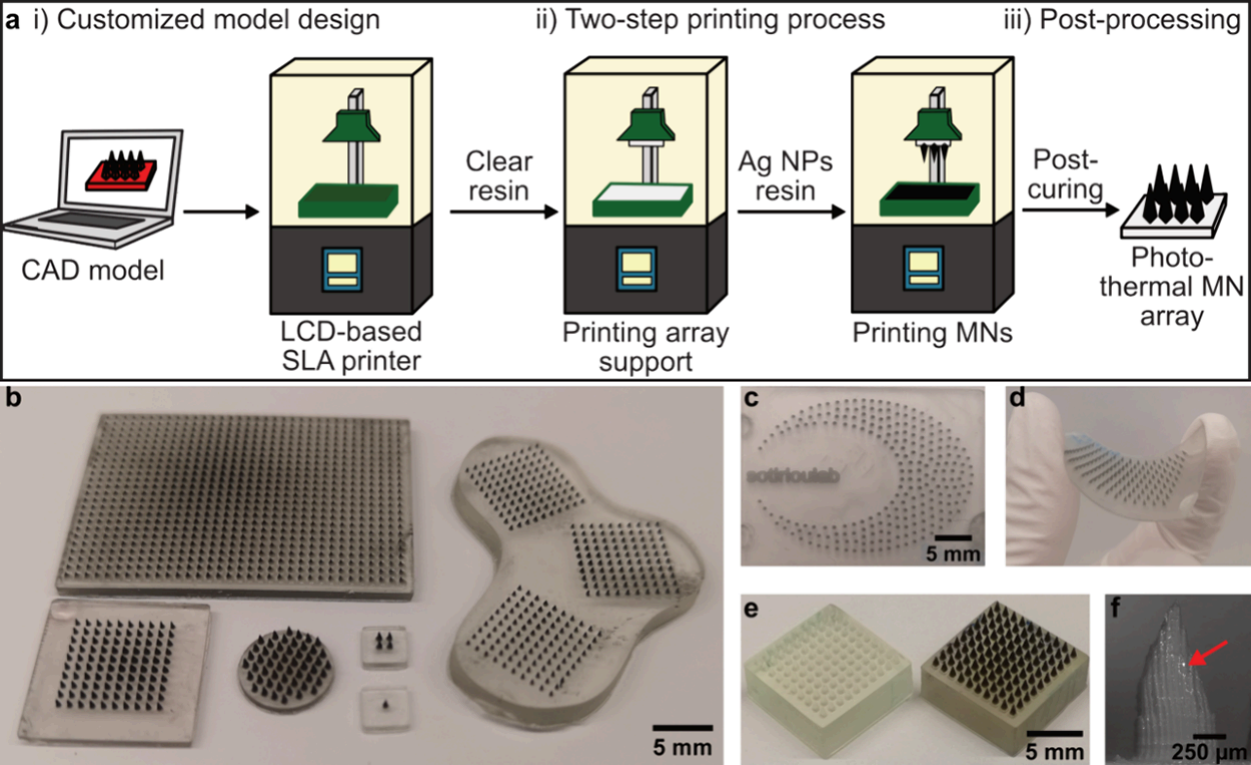
(a) Schematic illustration of the two-step 3D-printing of photothermal MN arrays by (i) uploading a customizable MN array design to an LCD–based SLA 3D-printer, (ii) printing the MN arrays in a two-step process in which the array support is printed using clear UV resin and the needles using a plasmonic Ag/SiO_2_ (2 wt % SiO_2_) NP-filled UV resin, and (iii) postcuring the MN array under UV light for 10 min. (b–e) Digital images of 3D-printed photothermal MNs arrays with various shapes (b,c), using a bendable, biocompatible support (d), and in comparison to a Ag/SiO_2_ NP-free MN array (e). (f) SEM image of single 3D-printed photothermal MN, red arrow indicating high-density elements such as Ag on the surface of the MN.

Figure 1 shows the customizable fabrication of 3D-printed MN arrays loaded with plasmonic, photothermal Ag/SiO_2_ (2 wt % SiO_2_) NPs. We chose Ag NPs as the photothermal agent because of their known strong plasmon resonance, effective photothermal light-to-heat conversion, large absorption and scattering cross sections, and inexpensive material costs.^31^ Furthermore, based on the previous work from our group that analyzed the plasmonic coupling in Ag nanoaggregates produced by flame spray pyrolysis, we selected 2 wt % SiO_2_ for the photothermal MNs due to the optimal extinction in the NIR region.^30^ The produced Ag/SiO_2_ nanoparticles used here have been thoroughly characterized and their NIR plasmonic photothermal properties have been confirmed previously.^30^ The structure of the Ag/SiO_2_ NPs was verified by transmission electron microscopy (TEM, Supporting Information (SI), Figure S1a,b), where multiple primary Ag/SiO_2_ NPs are clustered together into nanoaggregates having a Ag core and a thin SiO_2_ coating. Furthermore, the primary Ag particle size distribution and the interparticle distance are in line with our past results (SI, Figure S 1d,e). We also confirmed the previously reported optimized extinction in the NIR region via UV/vis spectroscopy for Ag/SiO_2_ dispersed in the printer resin used here (SI, Figure S1c).^30^

The printing procedure of the photothermal MN arrays is illustrated in Figure 1a. The MN arrays were produced by utilizing an LCD-based SLA printer on the basis of a sliced CAD model. The MN array was printed in 2–3 h in two steps using (i) UV-curable resin to print the MN array support followed by (ii) Ag/SiO_2_ NP-loaded resin to print the MNs. The final photothermal MN array was detached and cured under UV light for 10 min. First, we studied the effect of printer settings such as antialiasing, gray level, and image blurring on the morphology of the MNs and the difference between output and input height reduction (Supporting Information (SI), Table 1). We decided to continue with the printer settings set to the maximum level for the antialiasing and image blurring and the minimum setting for the gray value. Example images of various shapes and sizes of such printed photothermal MN arrays are shown in Figure 1b,c demonstrating the customizability of the arrays produced here. We also investigated the effect of varying needle designs on the final MN dimension and appearance (SI, Figure S2). The appearance of single MNs under bright-field microscopy is depicted in Figure S2a showing sharp needles for all tested input heights (1.5–2.1 mm). Upon measuring the output height and width of MNs as a function of their input height for three different *H*/*W* ratios (SI, Figure S2b–e), we found that the needle output height is 20–50% lower than the input height. Furthermore, both output height and width decrease with decreasing input heights. The output height reduction is most prevalent for large *H*/*W* ratios, which is in agreement with previous reports on SLA-printing of MNs.^27^ The discrepancy between the output and input height may be caused by (i) the tessellation process, (ii) the software settings, and (iii) resin shrinkage during curing. Miller et al. argued that geometrical differences in SLA-printed MNs are caused during the tessellation process by compromising the height of the printed part by the amount of the layer height,^32^ which we determined to contribute to ∼9% of height reduction in our printing. The additional MN height reduction may be explained with the antialiasing and blurring settings of the software, which resulted in various sublayers for each layer with decreasing pixels for higher layer and sublayer numbers. This may cause incomplete polymerization of the resin on the edges and tips of the MNs, similar to the process of hatch spacing induced MN height reduction in laser-based SLA printing.^27^ Finally, the resin may shrink during the last curing step, resulting in smaller needles than designed. However, overall the printing process is reproducible and in-line with the literature for printing of NP-free MNs, indicating that we can controllably print photothermal MNs of varying dimensions here.

To study whether other types of resins can be used to print photothermal MN arrays, we employed a commercial biocompatible UV-resin. This resin is tested according to ISO 10993–3 and 10993–10, showing no skin sensitization, genotoxicity, carcinogenicity, or reproductive toxicity. A digital image of a resulting biocompatible, photothermal MN array is depicted in Figure 1d. The thin support layer allowed for the flexibility of the MN array. Figure 1e shows digital images of MN arrays printed without (left) and with (right) the Ag/SiO_2_ NP resin for the MNs. The dark coloration of the MNs in the arrays printed with Ag/SiO_2_ NPs, in contrast to the translucent MNs when a clear resin was used, indicates the presence of Ag/SiO_2_ NPs only in the MNs. To further evaluate the presence of Ag/SiO_2_ NPs in the MNs, we performed SEM with a backscattered detector and a single needle is depicted in Figure 1f. Bright spots can be distinguished on the needles, indicating the presence of heavier elements such as metals; however, it appears that the Ag/SiO_2_ NPs in the MNs are incorporated into the resin needle almost completely. Such successful incorporation of plasmonic NPs in the needle matrix may be beneficial for biological applications as a reduced tissue-particle interface is advantageous in terms of nanotoxicology concerns.^33,34^ In fact, we evaluated the cell cytotoxicity of cells after exposure to substrates printed with the biocompatible resin and with incorporated NPs, confirming that the NP-free resin results in high cell viability of 93 ± 10% (SI, Figure S3a). Substrates containing NPs show a slight reduction in cell viability compared to NP-free substrates with a total cellular viability of 67 ± 11%. However, this cellular viability is comparable to cells exposed to dispersed Ag NPs in solution at a nominal 25-fold lower mass concentration, indicating the reduction of Ag NP toxicity when incorporating the NPs into the resin matrix (SI, Figure S3b). To the best of our knowledge, we show for the first time the printing of photothermal MNs in a single step, requiring only a few hours of fabrication and allowing for customizable MN patch shapes and sizes.

### 2.2 Mixing of Ag/SiO_2_ NPs in Printing Resin

**Figure 2:**
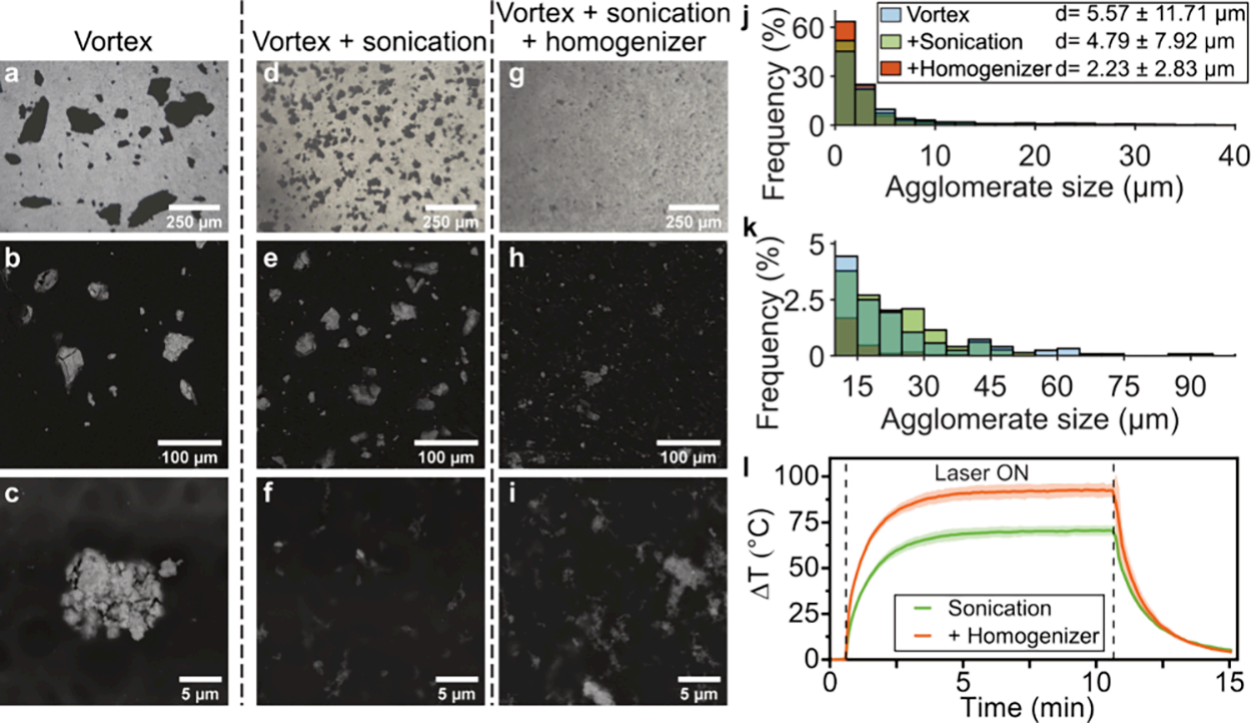
Influence of mixing methodology on Ag/SiO_2_ (2 wt % SiO_2_) NP dispersion in printing resin. Bright-field and SEM images for cured resin droplets with Ag/SiO_2_ NPs dispersed by (a–c) vortexing, (d–f) vortexing and ultrasonication, or (g–i) vortexing, ultrasonication, and bead homogenization. SEM-based agglomerate size distributions from 0–40 (j) and (k) 10–100 μm sizes with corresponding mean and standard deviation of the Feret diameter, bars plotted overlapping and partially transparent causing mixed coloration, *N* > 1000. (l) Photothermal response of MNs under NIR irradiation at 808 nm at 1 W cm^–2^ printed with Ag/SiO_2_ NP resin dispersed by vortexing and sonication or additional homogenization, *n* = 3.

We studied the dispersion of the Ag/SiO_2_ (2 wt % SiO_2_) NPs into the resin matrix using different mixing protocols such as vortexing, ultrasonication, and bead homogenization. To analyze the Ag/SiO_2_ NP agglomerate sizes in the resin after following the different dispersion protocols, we performed bright-field microscopy and SEM of cured droplets of Ag/SiO_2_ NP resin (Figure 2a–i). Vortexing alone of the Ag/SiO_2_ NP resin leads to the largest particle agglomerates with multiple particles above 100 μm as confirmed in the bright-field images (Figure 2a,d,g). Adding a step of sonication after vortexing improves the dispersion of the Ag/SiO_2_ NPs (Figure 2d–f). However, combining vortexing, sonication, and homogenization breaks down large particles, thus resulting in the smallest and most evenly distributed particle agglomerates in the resin as confirmed by SEM images (Figure 2b,c,e,f,h,i). This trend is also confirmed quantitatively when approximating the size of the agglomerates by measuring the Feret diameter of the pixels in the SEM images, as shown in the frequency plot in Figure 2j,k and by UV/vis analysis revealing higher extinctions for resins prepared with improved dispersion of Ag/SiO_2_ NPs (SI, Figure S4).

Bead homogenization results in high shear forces and bead collisions in the mixture,^35,36^ and it is therefore a critical step in the homogeneous dispersion and size reduction of the Ag/SiO_2_ NPs in the resin matrix. High shear mixing is known to reduce the agglomerate sizes for polymer nanocomposites.^37^ For example, Bensadoun et al. showed a 3-fold agglomerate size reduction of nanoclays in polyester resins when using high shear mixing compared to ultrasonication.^38^ Additionally, bead milling has been demonstrated to improve nanoclay dispersion and may result in smaller Ag/SiO_2_ NP sizes compared to high shear mixing.^39^ Finally, we analyzed the effect of the dispersion protocols on the final photothermal performance of the MN arrays by printing with a Ag/SiO_2_ NP resin mixed by sonication alone or in combination with bead homogenization. The temperature increase over time under laser radiation at 808 nm and 1 W cm^–2^ of such MN arrays is shown in Figure 2k. Combining both ultrasonication and homogenization to disperse the Ag/SiO_2_ NPs in the resin increases the maximum temperature difference of the MN arrays, further corroborating the improved homogenization of Ag/SiO_2_ NPs in resin for this combined protocol compared with ultrasonication alone. The improved dispersion of Ag/SiO_2_ NPs may improve the photothermal effect of the nanocomposites by (i) decreasing scattering losses and (ii) increasing the absorption. The scattering losses in the highly agglomerated nanocomposite samples may reduce the absorbance due to plasmonic coupling as Ag particles become closer at relatively low SiO_2_ spacer content resulting in the Ag/SiO_2_ NPs scattering light like larger particles.^40^ Meanwhile, an improved dispersion increases the availability of Ag/SiO_2_ NPs to interact with the laser light, thus increasing the total absorption of the composite. Importantly, we present here a straightforward dispersion protocol that can be easily employed and conducted in only a few minutes for the preparation of NP-resin composites, which stands in contrast to more complex NP modifications reported in literature for successful stereolithography printing.^41^

### 2.3 Mechanical Strength of Photothermal MN Arrays

**Figure 3:**
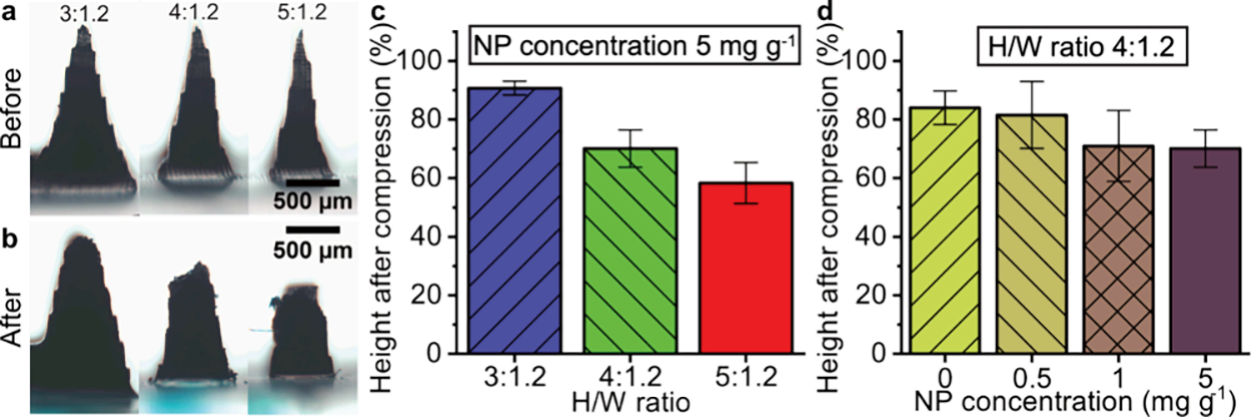
Height reduction of Ag/SiO_2_ (2 wt % SiO_2_) NP-loaded 3D-printed MN arrays after compression at 32 N for 30 s. Bright-field microscopy images of side-view of MN arrays for three height to base ratios (3:1.2, 4:1.2, and 5:1.2) before (a) and after (b) compression at Ag/SiO_2_ NP concentration of 5 mg g^–1^. Quantitative measurement of needle height reduction for (c) different dimensions of MN and (d) for various Ag/SiO_2_ NP loadings in resin for MN arrays at 4:1.2 height to base ratio. Data is shown as mean ± SD, *n* = 3.

To investigate the effect of MN geometry and Ag/SiO_2_ (2 wt % SiO_2_) NP loading on the mechanical strength of the MNs, we measured the height reduction of single MNs after compression with a texture analyzer for MN arrays of different *H*/*W* ratios and printed with varying concentrations of Ag/SiO_2_ NPs in resin. The compression was conducted for 30 s at 32 N to resemble the pressure applied with a thumb.^42^Figure 3a,b shows example microscopy images of photothermal MNs before and after compression for three *H*/*W* ratios. MN arrays with high *H*/*W* ratios are thinner, have sharper tips, and reduce more in height compared to those with a low *H*/*W* ratio. Figure 3c shows the height reduction of the MNs after compression for the different *H*/*W* ratios (for displacement-force curves, see SI, Figure S5). Increasing the *H*/*W* ratio leads to a greater height reduction, suggesting that needles with high *H*/*W* ratios are weaker against compression than needles with low *H*/*W* ratios, in agreement with the literature.^43^ Furthermore, the height reduction after compression of MN arrays with varying Ag/SiO_2_ NP concentrations in the resin is presented in Figure 3d. We observed a slight decrease in mechanical strength for increasing Ag/SiO_2_ NP loadings in contrast to previous studies showing an improved mechanical strength for increasing nanofiller concentrations in MNs.^16,44^ However, a direct comparison may not be suitable, as the factors affecting mechanical performance of nanocomposites are complex and depend on both particle–particle and particle–matrix interactions. As such, some nanocomposites have been reported to show increasing compression strength with increasing nanofiller fraction, while the compression strength of other nanocomposites only improves until an optimum volume fraction and decreases thereafter.^45^ Furthermore, in our process an increasing nanofiller concentration may interfere with the photocuring of the resin via light absorption of the Ag/SiO_2_ NPs, thus potentially reducing compression strength due to weak spots caused by incompletely cured resin. Additionally, when using the bead homogenizer as the dispersion tool, we observe microscale nanoaggregates, which may reduce the mechanical strength. However, upon evaluating the morphology and mechanical properties of MN arrays with a high NP loading of 5 mg g^–1^ after skin insertion and heating under NIR irradiation for 10 min, we observe the removal of intact needles from the skin and the MNs show the same mechanical strength against compression as MNs not used for intradermal photothermal heating (SI, Figure S5). Thus, the MN arrays developed here are sufficiently strong to pierce and heat the skin without loss of mechanical strength, and furthermore, the MN arrays could potentially be fit (after suitable disinfection) for repeated use with no loss of performance. Additionally, the photopolymer resins used here are (metha)crylate-based with water sorption values below 32 mg/mm^2^ (ISO standard) and a slow degradation profile. That minimizes any undesired nanoparticle release in the skin during their application.

### 2.4 Photothermal Effect of 3D-Printed MN Arrays

**Figure 4:**
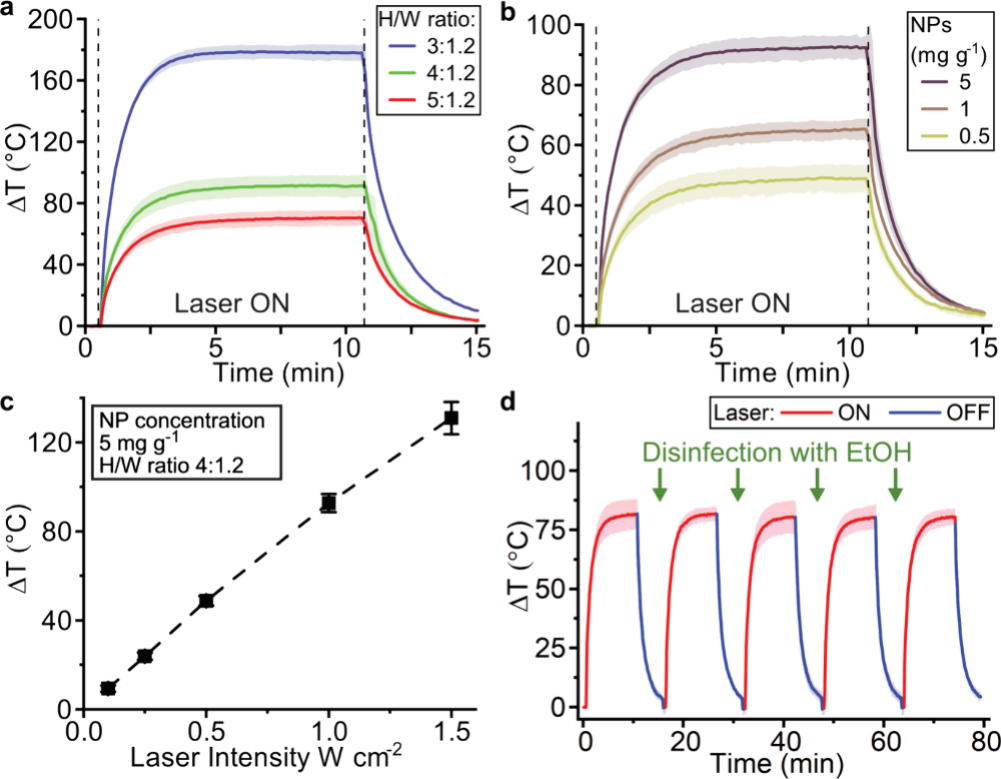
In air photothermal heating of 3D-printed Ag/SiO_2_ (2 wt % SiO_2_) MN arrays under laser irradiation at 808 nm at 1 W cm^–2^. (a) Average temperature increase of 10 × 10 pixels center point of MN arrays over time produced with different needle height to width ratios and (b) with varying Ag/SiO_2_ NP concentrations in the resin. (c) Maximum reached temperature increase (Δ*T*) of MN arrays as a function of the laser intensity. (d) Repeated heating of MN arrays with *H*/*W* ratio of 4:1.2 and Ag/SiO_2_ NP concentration of 5 mg g^–1^ after disinfection in EtOH. Data shown as mean ± SD, *n* = 3.

We explored the photothermal effect of the 3D-printed MN arrays under laser irradiation at 808 nm at 1 W cm^–2^ over time as a function of MN geometry and Ag/SiO_2_ (2 wt % SiO_2_) NP loading (Figure 4). First, we investigated the effect of *H*/*W* ratio on the temperature increase of MNs in air at room temperature, and the heat evolution over time is plotted in Figure 4a. Reducing the *H*/*W* ratio results in an increased maximum temperature difference (Δ*T*_max_) with Δ*T*_max_ reaching 108 °C when the *H*/*W* ratio is reduced from 5:1.2 to 3:1.2. Decreasing the *H*/*W* ratio while keeping the needle height constant leads to needles with a larger base diameter and volume, thus causing the improved photothermal response. On the other hand, keeping the same *H*/*W* ratio while increasing the needle height likewise increases the Δ*T* (SI, Figure S6). A similar trend was observed in which increasing the Ag/SiO_2_ NP concentration in the resin resulted in a larger Δ*T*_max_. The temperature increase over time for MNs arrays at *H*/*W* ratios of 4:1.2 for varying Ag/SiO_2_ NP concentrations (0.5–5 mg g^–1^) is depicted in Figure 4b. Increasing the Ag/SiO_2_ NP concentration by a factor of 10 results in an increase of Δ*T*_max_ by 44 °C. MNs arrays at *H*/*W* ratios of 4:1.2 and a Ag/SiO_2_ NP concentration of 5 mg g^–1^ reach Δ*T*_max_ values of 90 °C at laser intensities of 1 W cm^–2^. The temperatures achieved are well above both previously reported photothermal MNs^14,18,19^ and the required temperatures for photothermal therapies.^46^ To further investigate the minimum laser intensity required for detecting a photothermal effect of the 3D-printed MN arrays, we plot in Figure 4c the Δ*T*_max_ as a function of the laser intensity. Even at clinically acceptable laser intensities of 0.25 W cm^–2^, we reach a Δ*T*_max_ of 24 °C, which has been shown as a sufficient temperature increase for clinical photothermal treatments.^46^ Finally, to explore the reusability of the photothermal MN arrays produced here, we performed a cyclic heating under laser irradiation. We disinfected the MN arrays between every heating cycle in 70% ethanol for 5 min and recorded the temperature increase over time for a total of five cycles (Figure 4d). The 3D-printed MN arrays show photothermal stability over multiple rounds of disinfection, potentially allowing for repeated use of the MN arrays against bacteria.

**Figure 5:**
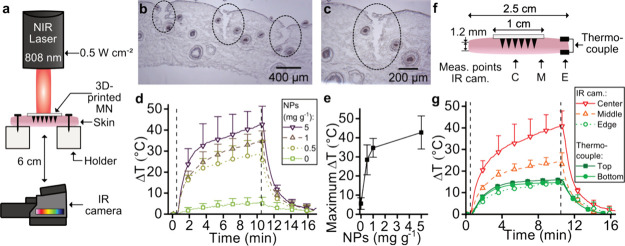
Skin insertion and in-skin hyperthermia of 3D-printed MN arrays. (a) Illustration of experimental setup to study in-skin hyperthermia from MN application under NIR. Microscopy images at (b) 5× and (c) 10× magnification of cross-section of porcine skin after MN array application. (d) In-skin hyperthermia over time under laser light irradiation at 808 nm at 0.5 W cm^–2^ by 3D-printed MN arrays produced with varying NP concentration in the resin. (e) Maximum temperature increase in the dermis as a function of the NP concentration in the resin. (f) Illustration of measurement points obtained from the thermal images of the IR camera at the center (C), middle (M), and edge (E) or from a thermocouple on the top epidermal or bottom dermal side of the skin sample. (g) Maximum temperature increase at the various measurement points across the skin sample. Data shown as mean ± SD, *n* = 3.

To confirm appropriate photothermal response in a clinically relevant environment, we studied the insertion capability and temperature increase of the 3D-printed, photothermal MN arrays in pork skin (Figure 5). Figure 5a shows the experimental setup for the measurement of intradermal hyperthermia. Thawed full-thickness pork skin of ∼1.2 mm thickness was secured on holders and imaged with a thermal camera from the dermal side. MNs arrays at *H*/*W* ratio of 4:1.2 and a needle height of ∼950 μm were inserted into the skin from the epidermal side. To study the insertion of MN arrays into the skin samples, we obtained cryosections of skin after removal of the MN arrays. Microscopy images of the tissue sections (Figure 5b,c) show successful breaching of the skin and penetration of the MNs to a depth of approximately 370 μm. Figure 5d shows the temperature increase over time of the skin under NIR irradiation (λ = 808 nm) of MN arrays at 0.5 W cm^–2^ produced with varying Ag/SiO_2_ NPs concentration. The Δ*T*_max_ as a function of the Ag/SiO_2_ NP concentration is plotted in Figure 5e. The laser irradiation of blank MN arrays alone causes a Δ*T*_max_ of 6 °C; however, the presence of Ag/SiO_2_ NPs significantly improves the temperature increase of skin, indicating successful intradermal photothermal heating of the 3D-printed MN arrays. Raising the Ag/SiO_2_ NP concentration 10-fold results in a Δ*T*_max_ of 14 °C, with a final intradermal temperature of 66 °C.

Bacterial skin infections can range from minor superficial infections such as impetigo and ecthyma to deeper cutaneous infections such as cellulitis and abscesses; therefore, to provide optimal efficiency of photothermal therapy, the heat distribution across the skin needs to be ensured.^47^ To study the heating profile from the MN arrays across the skin, we obtained the Δ*T*_max_ across various points of the skin sample measured from thermal images obtained with an IR camera and thermocouples as illustrated in Figure 5f. Figure 5g shows the average temperature increase over time for the various measurement points. At the center point of the area of the skin covered by the MN array, the average temperature increases the most with a Δ*T*_max_ of 41 °C. The temperature of the skin not directly in contact with the MN array increases to only a Δ*T*_max_ of 25 or 15 °C at ∼0.5 or 1 cm distance to the MN center, respectively (corresponding to measurement point “middle” or “edge” in Figure 5g). Furthermore, we observe that the full-thickness skin is evenly heated in the vertical direction with a difference in Δ*T*_max_ of less than 2 °C between the top and the bottom of the skin. Importantly, at the edge of the skin sample we obtain a therapeutically relevant temperature increase with Δ*T*_max_ of ∼15 °C. This temperature is in line with reported target temperatures for photothermal therapies,^46^ thus suggesting that the here reported MN arrays may be employed for therapies in the clinics even for deeper skin infections affecting the dermis.

### 2.5 Antibacterial Activity of 3D-Printed Photothermal MN Arrays

**Figure 6:**
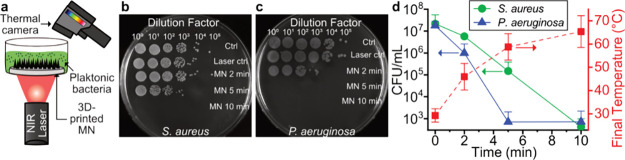
Antibacterial activity of photothermal 3D-printed MN arrays. (a) Schematic of experimental setup for evaluation of temperature profile and antibacterial effect of MN arrays in planktonic bacteria under NIR irradiation at 808 nm at 1 W cm^–2^. Top-view image of serial dilution on agar of (b) *S. aureus* and (c) *P. aeruginosa* for treatment with and without MN arrays and varying durations of laser irradiation (0, 2, 5, 10 min). (d) Bacteria quantification (CFU mL^–1^) of *S. aureus* (green circles) and *P. aeruginosa* (blue triangles) after continuous NIR irradiation and corresponding final temperature (right axis, red squares, *n* = 6). Data shown as mean and SD, *n* = 3.

Finally, we investigated the use of 3D-printed photothermal MN arrays as an antibacterial device. We chose two bacterial species, *S. aureus* and *P. aeruginosa*, as the model organisms based on their high association to skin infections.^48,49^ We printed the photothermal MN arrays to neatly fit into a cell culture plate (disk shape) and covered the arrays with a suspension of planktonic bacteria (Figure 6a). We irradiated the MN arrays with a laser (λ = 808 nm, 1 W cm^–2^) from the bottom, and the temperature increase was recorded with a thermal camera from the top. After varying laser irradiation durations (0–10 min), the bacterial suspension was cultured on growth agar overnight and the bacterial colony forming units quantified. Figure 6b,c shows representative top-view dark-field images of bacterial suspensions grown on agar for *S. aureus* and *P. aeruginosa*, respectively. Bacteria without any treatment (ctrl) or bacteria only exposed to laser irradiation for 10 min (laser ctrl) grew to a bacterial density of around 10^7^ CFU mL^–1^ which is suitable to reflect a bacterial skin manifestation.^50^

However, adding photothermal MN arrays under laser irradiation reduces the bacterial growth for both strains. The quantification of bacterial growth (left abscissa) and the final temperature attained (right abscissa) as a function of laser irradiation duration is shown in Figure 6d. The final temperature increases as a function of the time under laser irradiation and reaches ∼67 °C after 10 min of irradiation. As the temperature increases, the bacteria are killed and we observe a 4.7- and 4.4-log reduction in bacterial growth after 10 min of laser treatment for *S. aureus* and *P. aeruginosa*, respectively. Since we used Ag/SiO_2_ (2 wt % SiO_2_) NPs as the photothermal agent in the MN arrays, we confirmed by using an Ag ion meter that Ag^+^ ions were not released during the laser irradiation of 10 min. Therefore, intrinsic antibacterial activity of the Ag/SiO_2_ (2 wt % SiO_2_) NPs is not expected to contribute to the antibacterial effect observed here. Moreover, bacterial samples exposed to laser irradiation without MNs present or MNs alone without laser irradiation did not reduce the bacterial growth significantly (SI, Figure S7), indicating that the antibacterial effect observed here may mainly be caused by the photothermal heating. Finally, *P. aeruginosa* seems to be more sensitive to the temperature increase, with the maximum bacterial reduction obtained already after 5 min of treatment. This can be explained by the fact that *P. aeruginosa* is a Gram-negative bacterium that, as opposed to Gram-positive bacteria such as *S. aureus*, have a thin cell wall and are surrounded by an outer membrane rendering them more susceptible to heat-associated cell wall and membrane damage.^51^ The here-reported antibacterial performance is similar to previously reported antibacterial ablation of biofilms from plasmonic, photothermal films.^30^ Therefore, the 3D-printed MN arrays may be employed for antibacterial therapy.

## 3 Conclusions

In this work, we demonstrated the customizable manufacturing of photothermal MNs in a single 3D-print on a commercial low-cost SLA-printer. We fabricated photothermal MN arrays of varying size, shape, and needle geometry with the photothermal agent only present in the MNs. We optimized the dispersion of the photothermal agent in the printing resin and found that dispersion via sonication and bead homogenization improved the photothermal effect of the MN arrays. The photothermal MN arrays showed sufficient mechanical strength to pierce the skin and exhibited a high temperature increase under NIR-radiation at low laser intensities. Finally, we successfully reduced the bacterial growth of two bacteria commonly causing skin infections by irradiating the photothermal MN arrays with a NIR laser in bacterial suspensions. This work introduces for the first time a manufacturing protocol for photothermal MN arrays in a single 3D-print and lays the foundation for their clinical translation, providing the field with a low-cost fabrication method with high customizability and speed. Future studies will explore the *ex* and *in vivo* performance of such antibacterial photothermal MN arrays.

## 4 Experimental Section

### 4.1 Synthesis of Ag/SiO_2_ (2 wt % SiO_2_) Nanoparticles (NPs)

The plasmonic Ag/SiO_2_ (2 wt % SiO_2_) NPs were synthesized by flame spray pyrolysis. In short, 100 mL of solvent was prepared by mixing 1:1 acetonitrile (99.8%, Sigma-Aldrich) and 2-ethylhexanoic acid (2-EHA, 99%, Sigma-Aldrich) and dissolving silver acetate (99%, Alfa Aesar) to a concentration of 0.4 M under reflux at 100 °C. After cooling the solution to around 70 °C, 153 μL of hexamethyldisiloxane (HMDSO, ≥ 98%, Sigma-Aldrich) was added to achieve the target composition of 2 wt % SiO_2_. The liquid spray was ignited by a methane/oxygen support flame with flow rates of 1.5 and 3.2 L min^–1^, respectively. Thereafter, the dissolved precursor was dispersed at a rate of 5 mL min^–1^ into a fine spray by oxygen gas flowing at 5 L min^–1^. With the aid of a vacuum pump the particles were filtered (250 μm pore size, Hahnemühle) and collected as a powder.

### 4.2 3D-Printing of Photothermal MNs

Photothermal MNs were fabricated by using a two-step method, utilizing a liquid-crystal display (LCD)-based stereolithography (SLA) 3D printer (Anycubic Photon, Anycubic) and two types of resin. A smaller vat was custom-made and printed into a bigger vat to reduce resin waste. CAD software (Fusion 360, Autodesk) was used to design the MN patches, and the slicing software (Chitubox 64, CBD-tech) translated the CAD design into layers of 0.01 mm. The print was started by printing the MN array support layer with a clear photopolymer (Value UV/DLP Resin STANDARD, PrimaCreator) or a biocompatible photopolymer (GR-10, pro3Dure; biocompatibility assessed according to ISO 10993–3 and 10993–10). A photothermal resin was prepared for the MNs by mixing 5 mg g^–1^ Ag/SiO_2_ (2 wt % SiO_2_) NPs with clear resin through vortexing. Afterward, the mixture was homogenized using bead homogenization (Lysing matrix z, MP Biomedicals) five times for 1 min at 6 m s^–1^ with cooling on ice for 1 min between cycles using a homogenizer (FastPrep-24 5G, MP Biomedicals) followed by 15 min of sonication in a bath sonicator. The Ag/SiO_2_ NP-resin was used to print photothermal MNs onto a clear support layer. When finished, the MN patch was rinsed in 2-propanol to remove excess resin followed by 10 min curing under UV light. For imaging of Ag/SiO_2_ NP resins, 10 μL droplets of resin were cured under UV-light and imaged with a bright field microscope. Scanning electron microscopy (SEM, Phenom Pharos, Thermo Fisher Scientific) was performed on cured-resin droplets with internal surface revealed by cutting off cryosections. The size distribution of agglomerates was measured setting a pixel threshold in ImageJ (US National Institute of Health) and analyzing the Feret diameter of two SEM images at different magnifications, *N* > 1000.

### 4.3 Cell Viability Assay

A standard cell viability test using the human A549 cells (lung adenocarcinoma epithelial cell line) (ATCC CCL-185) and MGA-U343-GFP cells cultured in Dulbecco’s modified Eagle’s medium DEM (GIBCO) with 10% fetal bovine serum (GIBCO) and 50 U/mL penicillin–streptomycin (ThermoFischer) was performed as described in detail previously.^52^ The cells were grown at 37 °C and 5% CO_2_ until 90% confluency and 15,000 cells were incubated for 16 h before washing with PBS and exchange of medium. Test substrates were printed with NP-free or NP-resin to fit into well-plates to be covered by cell medium to provide indirect contact to the cell layer. The cells were seeded by incubating 355 μL of medium containing 30,000 cells for 16 h. Afterward, the cells were washed and submerged in medium, and the test substrates were exposed to the cell culture. After 24 h incubation, the substrates were removed, resazurin was added, and the mixture was incubated for another 4 h. 200 μL of the medium was transferred to a 96-well plate, and fluorescence was measured using luminescence spectrometer (Tecan Spark Cyto) with excitation and emission wavelengths at 540 and 590 nm, respectively. The experiments were conducted in biological triplicate, and mean ± standard deviation was plotted.

### 4.4 Mechanical Testing of 3D-Printed MNs

Mechanical testing was done using a texture analyzer (TA.XT plusC, Stable micro system). MN patches designed with four needles per patch (MNs height = 2 mm, height-to-width ratio (*H*/*W*) of 3, 4, or 5:1.2) were mounted onto the cylinder probe using double adhesive tape and pressed against the platform for 30 s at 32 N. The cylinder probe was run at 1 mm s^–1^ pretest, 1 mm s^–1^ test, and 1 mm s^–1^ post-test speed. Microscopy images of the MNs were taken before and after the compression and measured using ImageJ (US National Institute of Health).^53^

### 4.5 Insertion Capabilities of 3D-Printed MN Arrays

The skin of stillborn piglets was obtained from a local breeder and stored at −20 °C (ethical permit not required, according to the Swedish Board of Agriculture). Full-thickness skin was thawed in phosphate buffered saline (PBS, 0.1 M, pH 7.4) at room temperature. Afterward, a photothermal MN array (MNs input height = 2 mm, H/W = 4:1.2) was pressed into the skin for around 1 min with thumb pressure. The skin sample was embedded in Optimal Cutting Temperature medium (OCT, VWR) and frozen at −80 °C. 10 μm cryosections were collected, and the skin sections were imaged.

### 4.6 Analysis of In-Air and Intradermal Photothermal Effect

The photothermal effect of the MNs was measured using an 808 nm fiber-coupled diode laser with a diffuser (Laser Century), resulting in a squared laser area and a shutter to turn it on and off digitally. The power and distance were altered to achieve the desired intensity (0.1–2.0 W cm^–2^), and a thermal imaging camera (testo 871, Testo accuracy ±2 °C, sensitivity <0.08 °C) measured the temperature increase. When measuring in air, the laser and the thermal imaging camera were placed above the MN patch. For intradermal measurements, the MN arrays (MN height = 2 mm, *H*/*W* = 4:1.2) were pressed into thawed porcine skin using thumb pressure for 30 s and irradiated by the laser through the support layer for 10 min. The thermal imaging camera was placed underneath the skin facing the dermal side. The thermal images were analyzed for an average of 10 × 10 pixels around the selected measurement point. Furthermore, a thermometer with two thermocouples of type K (SDL200, Extech Instruments) was used to record the temperature every 10 s on the epidermal skin side next to the MN arrays and on the dermal side.

### 4.7 Antibacterial Studies

The antibacterial studies were carried out using *S. aureus* (ATC 25 923) and *P. aeruginosa* (PA01). Liquid cultures were prepared by inoculating luria broth (LB) from frozen bacterial stocks and incubating the mixture overnight at 37 °C. MN patches (MN height = 2 mm, *H*/*W* = 4:1.2) with a particle loading of 5 mg g^–1^ were printed on a round support to precisely fit one well in a 48-well plate and placed with the needles facing up. 200 μL of bacterial suspension (OD_600 nm_ = 0.1) was added to the MNs and exposed to laser irradiation (1 W cm^–2^) for 10 min. Laser controls were performed with the same laser exposure without MNs, and MN controls were performed with MNs but without exposure to laser irradiation. The bacteria were diluted with PBS in 1:10 series. 10 μL of dot spots of all the dilutions and 100 μL of selected dilutions were added on LB agar plates to have 3–300 colonies. The plates were incubated at 37 °C and colonies were counted the next day.

## Supporting Information Available

Additional characterization of the developed materials and devices (PDF)
