# Supplementary material for: Customizable Fabrication of Photothermal Microneedles with Plasmonic Nanoparticles Using Low-Cost Stereolithography Three-Dimensional Printing
Source: ACS Appl Bio Mater. 2024 Jun 15;7(7):4533–41. doi: 10.1021/acsabm.4c00411 (PMC11253096; doi:10.1021/acsabm.4c00411)
Supplement: Supplementary file 1 — mt4c00411_si_001.pdf [file mt4c00411_si_001.pdf]

Supporting Information

Customizable fabrication of photothermal microneedles with plasmonic nanoparticles using low-cost stereolithography 3D-printing

*Jill Ziesmer, Isabel Sondén, Justina Venckute Larsson, Padryk Merkl, Georgios A. Sotiriou**

Department of Microbiology, Tumor and Cell Biology, Karolinska Institutet, SE-171 77, Stockholm, Sweden

*E-mail: georgios.sotiriou@ki.se

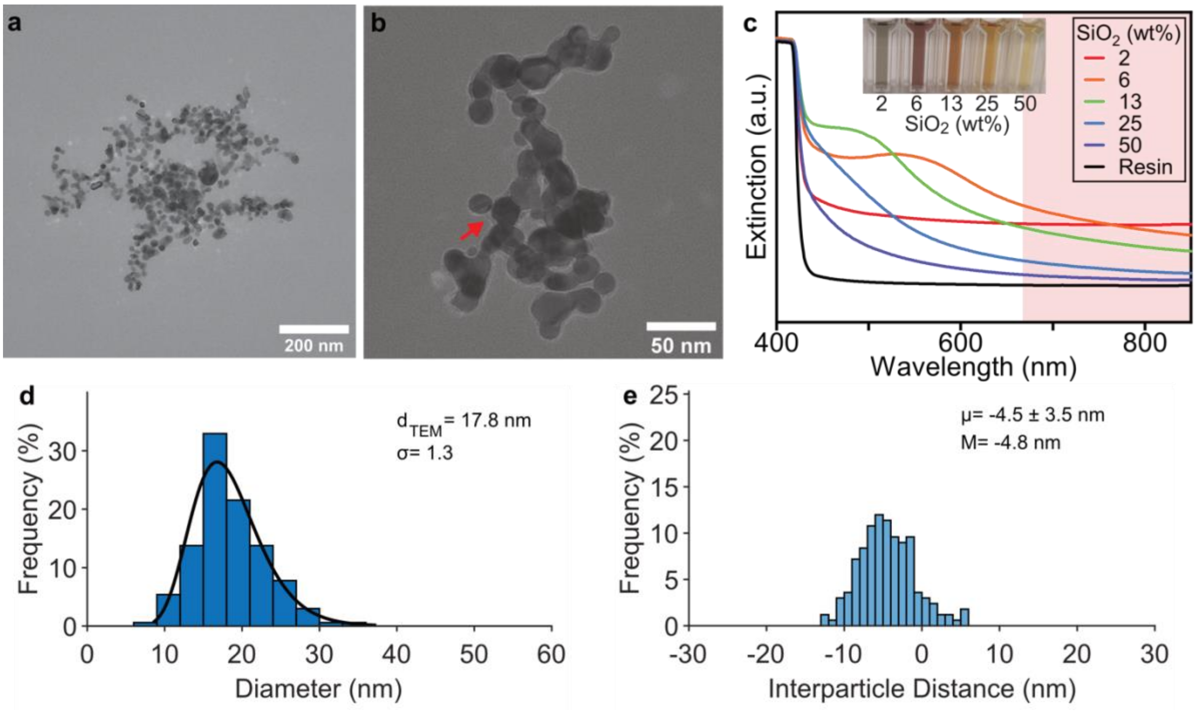

Figure S1: (a-b) Transmission electron microscopy (TEM) images of Ag/ SiO_2 (2 wt% SiO_2) nanoaggregates in suspension, red arrow indicates the SiO_2 coating**.** (c) UV/Vis spectra of resin dispersed with homogenization of Ag/SiO_2 NPs with varying SiO_2 content. NPs were dispersed at 0.05 mg g^{-1} and inserts show resin samples in cuvette. (d) TEM-based size distribution and (e) interparticle distance of primary NP of Ag/ SiO_2 (2 wt% SiO_2) nanoaggregates with geometric mean, standard deviation and modeled with a log-normal distribution function or with mean, standard deviation and median (M), respectively. N>100.

Table S1: Influence of the different printing settings on the final MN output per input height and an example bright field image of the MNs. The printer setting anti-aliasing, grey level and image blur were set on different values of their respective ranges. **Scale bar indicates 1 mm.**

No.	Anti-aliasing (2, 4, 6, 8)	Grey level (0 – 8)	Image blur (0, 2, 3, 4)	Output/ input height (%)	Example image
1	**0**	**0**	**0**	39.2	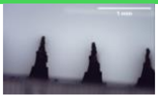
2	**8**	0	0	50.9	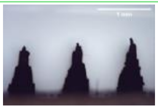
3	**2**	8	0	45.7	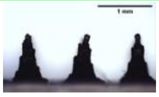
4	**2**	0	4	55.8	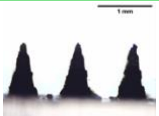
5	**8**	0	4	62.4	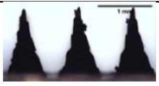
6	**2**	4	4	70.2	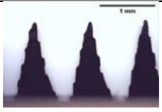
7	**2**	8	4	68.9	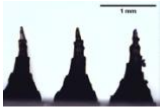
8	**4**	0	2	55.8	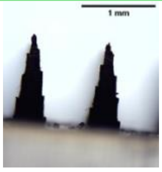
9	**8**	8	4	70.1	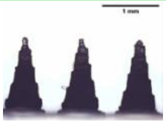

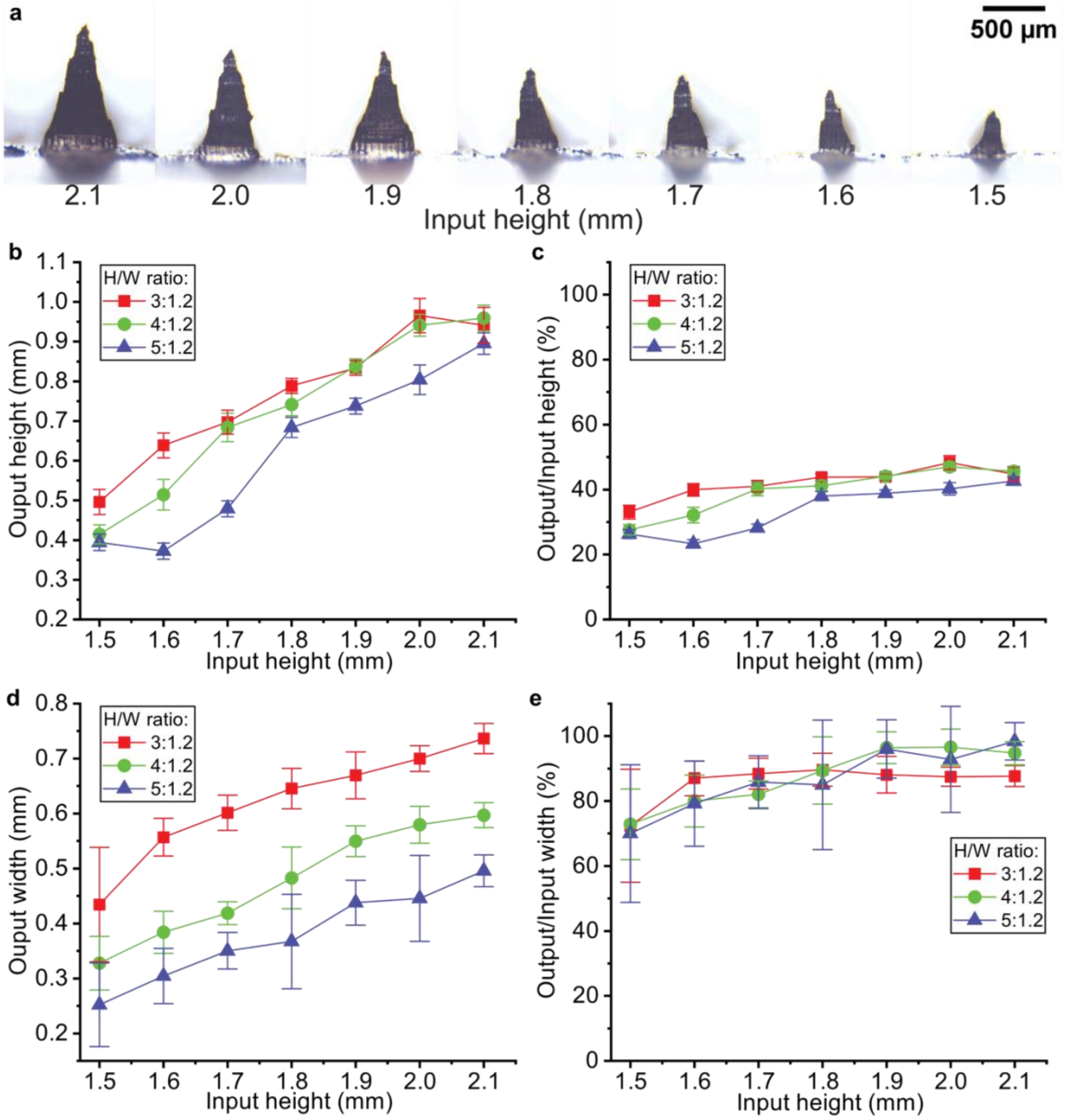

Figure S2: Investigation of needle dimensions after printing of Ag/SiO_2 (2 wt% SiO_2) NP filled MN arrays. (a) Side-view microscopic images of single photothermal 3D-printed needles for aspect ratio of 4:1.2 at different input heights. (b) Output needle height and (c) relative output needle height per input needle height as a function of input height for three height to width ratios. (d) Output needle width and (e) relative output needle width per input needle width as a function of input height for three height to width ratios. Data plotted as mean ± SD, n=2.

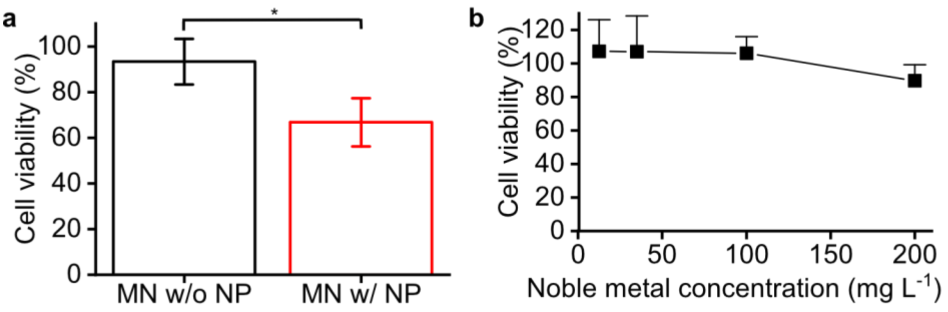

Figure S3: Cell viability after incubation with Ag NPs using resazurin assay. (a) Cell viability after incubation of cells to 3D-printed substrates with or without NPs dispersed in the printed resin. The particle concentration in the polymer resins was 5mg/g. (b) Cell viability after incubation of cells with dispersed Ag/SiO_2 (2wt% SiO_2) NPs in solution for increasing particle concentrations (corresponding to 12.5 – 200 mg L^{-1} noble metal (Ag) concentration).

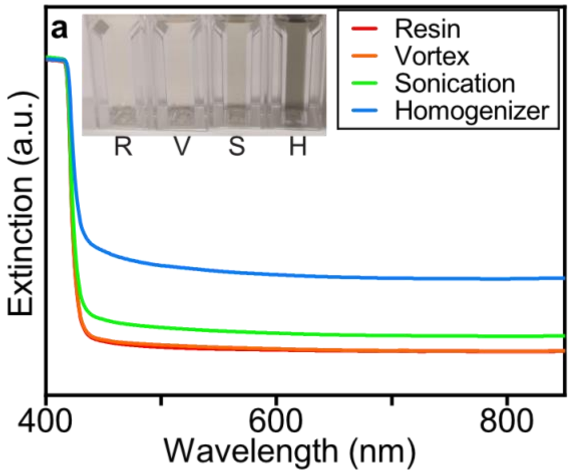

Figure S4: UV/Vis spectra of Ag/SiO_2 (2 wt% SiO_2) nanoaggregates in printing resin. (a) UV/Vis spectra of pure resin (R) or resin with Ag/SiO_2 NPs after dispersion with vortexing (V), sonication (S) or additional homogenization (H). NPs were dispersed at 0.05 mg g-1 and inserts show resin samples in cuvette.

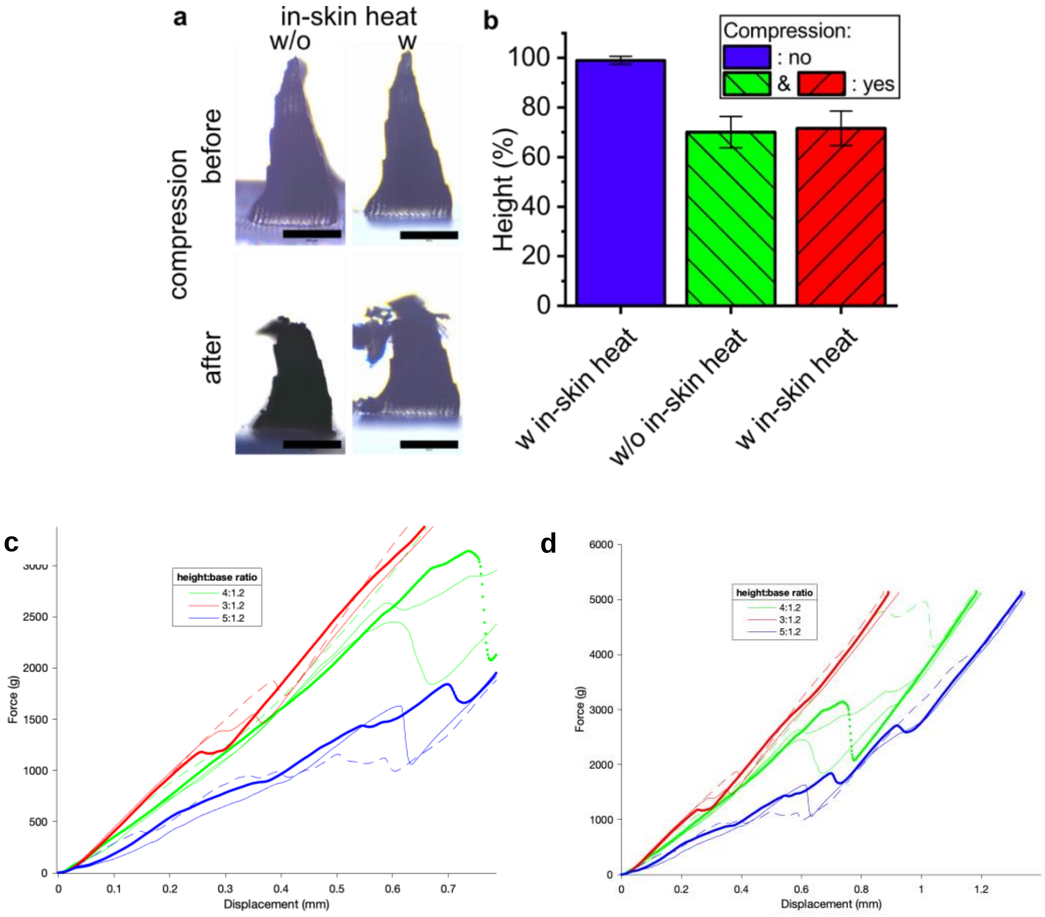

Figure S5: Height reduction of NP-loaded 3D-printed MN arrays (5 mg g^{-1}, H/W 4:1.2, Hinput = 2 mm) after skin insertion and NIR irradiation at 808 nm for 10 min. (a) Bright-field microscopy images of side-view of MNs for before and after compression at 32 N for 30 sec of MNs used without (w/o) or with (w) intradermal heating. (b) Quantitative measurement of needle height reduction for MNs after intradermal heating and for MNs after compression. Data is shown as mean ± SD, n=3. (c) and (d) Displacement-Force graphs of the different MN arrays produced here.

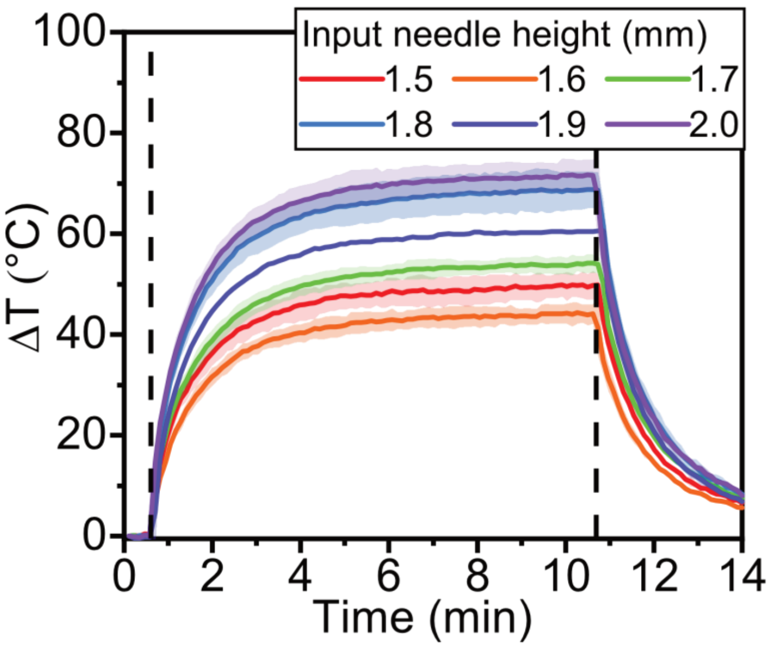

Figure S6: Temperature increase of 3D-printed photothermal MNs with different input heights at H/W ratio of 4:1.2 and 5 mg g^{-1} Ag/SiO_2 (2 wt% SiO_2) NP loading, data plotted as mean ± SD, n=2.

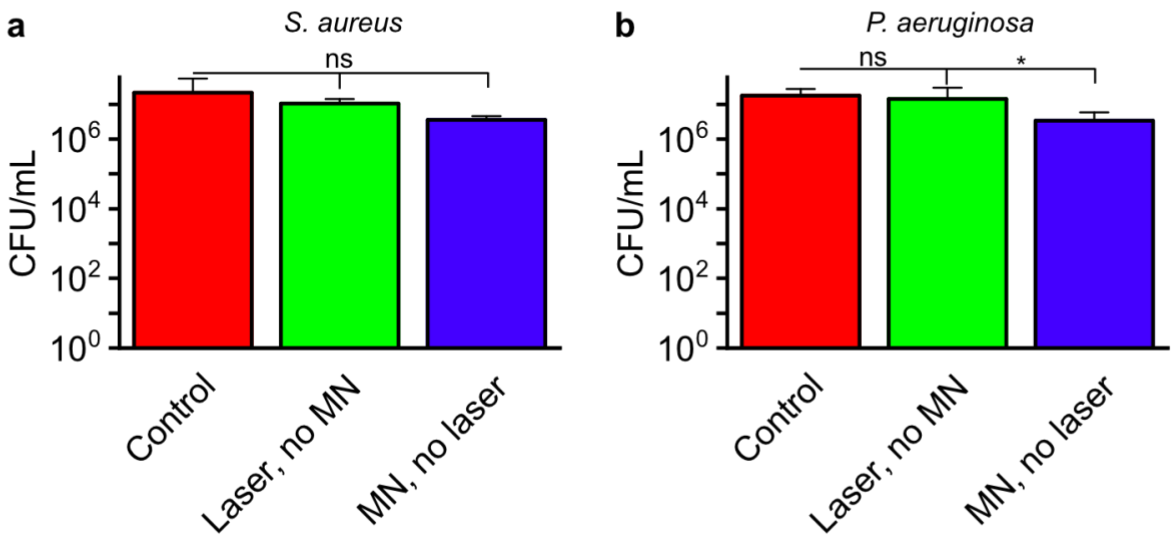

Figure S7: Bacteria quantification (CFU mL^{-1}) of controls for antibacterial experiment with (a) *S. aureus* and (b) *P. aeruginosa*. Controls were untreated planktonic bacteria (control), bacteria exposed to NIR irradiation for 10 min at 808 nm (laser, no MN), or bacteria exposed to MNs without laser irradiation for 10 min (MN, no laser). Data shown as mean + SD, statistical significance analyzed with one-way ANOVA and Turkey test, significance level of 0.05, ns = non-significance, asterisk indicates p-value < 0.05.
